# Outcome in paraplegic dogs with or without pain perception due to thoracolumbar fibrocartilaginous embolic myelopathy or acute non-compressive nucleus pulposus extrusion

**DOI:** 10.3389/fvets.2024.1406843

**Published:** 2024-05-09

**Authors:** Go Togawa, Melissa J. Lewis, Dillon Devathasan

**Affiliations:** ^1^Department of Veterinary Clinical Sciences, College of Veterinary Medicine, Purdue University, West Lafayette, IN, United States; ^2^Department of Clinical Sciences, College of Veterinary Medicine, Auburn University, Auburn, AL, United States

**Keywords:** fibrocartilaginous embolism, ischemic myelopathy, missile disc herniation, paralysis, canine, deep pain negative

## Abstract

**Background:**

Fibrocartilaginous embolic myelopathy (FCEM) and acute non-compressive nucleus pulposus extrusion (ANNPE) are common causes of acute spinal cord injury in dogs. Outcome among paraplegic deep pain positive (DPP) and deep pain negative (DPN) dogs with either condition and factors influencing recovery have not been clearly established.

**Methods:**

Dogs with thoracolumbar FCEM or ANNPE resulting in paraplegia presenting to university hospitals between 2012 and 2022 were retrospectively included. Diagnosis of FCEM or ANNPE was based on clinical and magnetic resonance imaging findings. Outcome was defined as successful (recovery of independent ambulation) or unsuccessful (non-ambulatory ≥3 months following diagnosis or at the time of death/euthanasia). Logistic regression analysis was performed to investigate associations between clinical or imaging variables and outcome.

**Results:**

Thirty-one dogs were included. In total, 14 dogs were initially paraplegic DPP (8 FCEM, 6 ANNPE) and 17 dogs were paraplegic DPN (11 FCEM, 6 ANNPE). Outcome was available for 26 dogs (14 DPP, 12 DPN) with a median follow-up time of 182 days (range 0–2,311) including 2 dogs euthanized at the time of diagnosis; 1 of 12 DPN dogs (8.3%) regained independent ambulation, whereas 9 of 14 DPP dogs (64.3%) regained independent ambulation. DPN dogs had a significantly higher risk of not regaining independent ambulation compared with DPP dogs (OR: 47.40, 95% CI: 2.09–1073.99). No other variables were associated with outcome.

**Conclusion:**

While recovery of ambulation was possible, these results confirm that the absence of pain perception is a useful negative prognostic indicator in dogs with severe thoracolumbar FCEM or ANNPE.

## Introduction

Fibrocartilaginous embolic myelopathy (FCEM) occurs when fibrocartilaginous material presumed to originate from the nucleus pulposus of an intervertebral disc that infarcts the spinal cord vasculature, resulting in ischemic necrosis of a specific region of spinal cord parenchyma (1). Acute non-compressive nucleus pulposus extrusion (ANNPE) is characterized by the herniation of non-degenerated nucleus pulposus material from an intervertebral disc, causing a contusive, non-compressive or minimally compressive injury to the spinal cord (2). While definitive diagnosis requires histopathologic examination, antemortem diagnostic criteria exist to differentiate between FCEM and ANNPE (3, 4), and these two conditions are commonly grouped together given their clinical similarities. Overlapping clinical features of presumptive FCEM and ANNPE include peracute or acute onset of non-progressive spinal cord dysfunction typically associated with exercise or minor trauma, lacking persistent spinal pain (3–13). While both conditions generally have a favorable prognosis, limited information exists regarding the recovery of walking or continence in severely affected dogs with FCEM or ANNPE (3–13).

Perception of a painful stimulus (often simply referred to as ‘pain perception’) is an important prognostic indicator for thoracolumbar intervertebral disc extrusion (TL-IVDE) with approximately 60% of surgically managed dogs without pain perception recovering the ability to walk (14). Across previous studies that include paraplegic dogs with or without pain perception diagnosed with FCEM or ANNPE, recovery rates for walking vary from 45 to 100% for dogs with pain perception and 0 to 75% for dogs without pain perception (3, 7–9, 12, 13). While paraplegic dogs without pain perception secondary to FCEM or ANNPE are generally presumed to have a worse prognosis, the high rate of euthanasia around the time of diagnosis and small number of severely affected dogs included in most studies confound this conclusion (3, 7–9, 12, 13). The outcome in paraplegic dogs with or without pain perception secondary to FCEM or ANNPE and factors influencing their prognosis have not been clearly established.

The aim of this study was to describe the clinical features and outcome of paraplegic dogs with or without pain perception secondary to presumptive thoracolumbar FCEM or ANNPE. Our secondary goal was to investigate if any clinical parameters could predict outcome in these dogs. We hypothesized that paraplegic dogs without pain perception would have a poor outcome compared with paraplegic dogs with intact pain perception, and that specific clinical variables could aid in differentiating between successful and unsuccessful outcomes in severely affected dogs with presumptive thoracolumbar FCEM or ANNPE.

## Materials and methods

### Study design and inclusion criteria

The medical record databases of the Purdue University Veterinary Hospital and Auburn University Veterinary Clinic from September 2012 to July 2022 were retrospectively searched for dogs diagnosed with FCEM or ANNPE. Search terms included fibrocartilaginous embolism or embolic myopathy, FCE, FCEM, spinal cord stroke, ischemic myelopathy, acute non-compressive (or noncompressive) nucleus puposus extrusion, ANNPE, and missile disc (or disk), traumatic disc, high velocity low volume disc, and type III disc herniation. To be included, dogs had to have a presumptive diagnosis of FCEM or ANNPE with neurolocalization between the third thoracic to third lumbar (T3–L3) or fourth lumbar to third sacral (L4–S3) spinal cord segments, resulting in paraplegia with or without pain perception on the initial neurological assessment. Presumptive diagnosis of FCEM or ANNPE was based on MRI features according to previously published descriptions (3, 4, 6–8, 15–17). Briefly, findings compatible with a diagnosis of ANNPE could include a focal, non-longitudinal, intramedullary T2W hyperintensity overlying an intervertebral disc; an affected intervertebral disc space that was narrowed, had reduced volume of the residual nucleus pulposus, or a cleft in the dorsal part of the annulus fibrosus; extradural material compatible with hydrated nucleus pulposus causing no or minimal spinal cord compression; and if T1W fat-suppressed post-contrast images were acquired, focal meningeal/epidural contrast enhancement (3, 4, 8, 15). The MRI findings supportive of a diagnosis of FCEM could include a focal, well-demarcated, longitudinal, T2-hyperintense intramedullary lesion primarily affecting the gray matter and the absence of MRI findings supportive of ANNPE (3, 4, 6, 7, 15–17). Cerebrospinal fluid analysis was not required for inclusion.

### Clinical and imaging data

Data obtained from the medical record included age, sex, breed, body weight, and body condition score and details regarding onset, examination findings, diagnosis, treatment, and outcome. Onset was based on owner recollection and categorized as peracute or acute. Peracute onset was defined as progression from normal ambulation to paraplegia in <1 h, and acute onset was defined as progression from normal ambulation to paraplegia between 1 and 24 h. Time from the onset of neurologic signs to presentation was estimated from the medical record and recorded in hours. Onset duration was then defined as <12 h, 12–24 h, >24 h. Initial neurological examination findings were recorded including pelvic limb and tail pain perception status and the presence of spinal shock or Schiff–Sherrington posture. Spinal shock was defined as depressed or absent segmental reflexes and/or muscle tone caudal to a lesion located between the T3–L3 spinal cord segments (18, 19). The dogs were classified as having spinal shock if they met the aforementioned definition on the intake neurological examination and their lesion on MRI did not affect the L4–S3 spinal cord segments. Schiff–Sherrington posture was defined as persistent, severely increased extensor tone of the thoracic limbs in most postures with a normal thoracic limb gait except for mild stiffness (20). The dogs were classified as having Schiff–Sherrington posture if they met this definition in the thoracic limbs on the intake neurological examination. Absent pain perception [deep pain negative (DPN)] was defined as lack of an overt, repeatable behavioral response to pinching over the bone of the medial and lateral digits of the pelvic limbs and coccygeal vertebrae using hemostats or needle drivers. Dogs were classified as DPN when pain perception was absent in both of the pelvic limbs and tail (if information regarding tail was provided). If pain perception was present in any one of the toes or tail but absent elsewhere, the dog was classified as having pain perception [deep pain positive (DPP)]. The status of urinary or fecal continence was noted when this information was documented.

All dogs had an MRI of the thoracolumbar spine using a 1.5 T (GE Signa LXi) or 3.0 T magnet (Phillips Infineon or Siemens Magnotome Skyra). In all dogs, sagittal and transverse T2W, sagittal and transverse T1W, and sagittal STIR sequences were performed. Sagittal, transverse, dorsal post-contrast T1W, sagittal half-Fourier single-shot turbo spin echo (HASTE), and dorsal and/or transverse gradient echo sequences were variably obtained. Based on MRI, the lesion location was categorized as affecting T3–L3, L4–S3, or involving both spinal cord regions. Using commercially available software, OsiriX (OsiriX Foundation, Geneva, Switzerland), lesion dimensions were quantified in all dogs as previously described. On T2W sagittal images, the lesion length to vertebral length (LL:VL) ratio was obtained by measuring the longitudinal extent of intramedullary hyperintensity as a ratio of the length of the second lumbar vertebral body. Using T2W transverse images, the percentage cross-sectional area of the lesion (PCSAL) was calculated by measuring the largest area of intramedullary hyperintensity as a percentage of the total cross-sectional area of the spinal cord at the same level (7, 8).

Supportive care treatments were recorded. For all dogs, basic at-home rehabilitation exercises were recommended until dogs were able to walk independently and included massage, passive range of motion, assisted standing, and assisted walking. Participation in a formal outpatient physical rehabilitation program after discharge was noted including duration of such therapy, if known.

### Outcome and follow-up

Outcome was defined as successful or unsuccessful and was determined based on details in the medical records and follow-up telephone conversations with owners or referring veterinarians. A successful outcome was defined as recovery of independent ambulation (the ability to take ≥10 consecutive unassisted steps at a time). An unsuccessful outcome was defined as being non-ambulatory at the time of last follow-up obtained at least 3 months after diagnosis. Dogs that died or were humanely euthanized were categorized as having an unsuccessful outcome if they were still non-ambulatory due to their original injury at the time of death or euthanasia. The reason of death or euthanasia was recorded when available. Outcome was classified as unknown for dogs lost to follow-up or where available follow-up information (other than death or euthanasia) was less than 3 months from the time of injury. Due to limited information available, the status of urinary or fecal continence was not included in the definition of outcome (successful or unsuccessful).

### Statistical analysis

All statistical analyses were performed using commercially available software, STATA (STATE SE v.17.1, StataCorp, College Station, TX). For descriptive statistics, continuous variables were examined for normality using the Shapiro–Wilk test and reported as median (range) or mean ± standard deviation, as appropriate. Categorical variables were presented as proportions. Logistic regression was performed to investigate associations between clinical or imaging variables and outcome (successful or unsucessful). Dogs classified as having an unknown outcome were not included in the outcome analysis. The following factors were included in the model: age (years), body weight (kgs), sex, body condition score, pain perception (Y/N), neurolocalization (T3-L3 vs. L4-S3), presence of spinal shock (Y/N/NA), presence of Schiff–Sherrington posture (Y/N), diagnosis (FCEM or ANNPE), LL:VL ratio, PCSAL, onset (per-acute or acute), duration prior to presentation (<12 h, 12–24 h, or >24 h), duration of hospitalization (days), and participation in a formal outpatient physical rehabilitation program (Y/N). Variables with values of *p* < 0.2 on univariate analysis were then included in multivariable regression analysis. *p*-values of less than 0.05 were considered significant.

## Results

### Study population and clinical and imaging data

Thirty-one cases met the inclusion criteria. 19 dogs were diagnosed with a presumptive FCEM, and 12 dogs were diagnosed with a presumptive ANNPE. 17 dogs were classified as DPP and 14 dogs were classified as DPN. Table 1 compares the clinical and imaging variables by presumptive diagnosis and pain perception status. The mean age at diagnosis was 6.3 ± 2.4 years. The mean body weight was 27.5 ± 13.3 kg, and the median body condition score was 5 (4–9). There were 16 males (5 sexually intact and 11 neutered) and 15 females (all 15 spayed). Breeds represented included the Labrador Retriever (*n* = 7), mixed breed dog (*n* = 7), Siberian Husky (*n* = 2), American Pit Bull Terrier (*n* = 2), Staffordshire Bull Terrier (*n* = 2), and 1 each of English Bulldog, American Eskimo, German Shepherd Dog, Border Collie, Whippet, Bernese Mountain Dog, Lagotto Romagnolo, Standard Poodle, Australian Shepherd, Affenpinscher, and Miniature Schnauzer. The primary presenting complaint in all dogs was paraplegia. 21 dogs had a per-acute onset, whereas the remaining 10 dogs had an acute onset. Onset duration was <12 h in 20 dogs, 12–24 h in 9 dogs, >24 h in 1 dog, and was unknown in 1 dog.

**Table 1:** Comparison of clinical and imaging variables based on presumptive diagnosis (FCEM vs. ANNPE) or pain perception status (positive vs. negative).

Variables	Diagnosis		Pain perception status	
	FCEM (*n* = 19)	ANNPE (*n* = 12)	Positive (*n* = 17)	Negative (*n* = 14)
Pain perception (positive/negative)	11/8	6/6		
Mean age (y)	6.3 ± 2.7	6.2 ± 2.0	6.8 ± 2.8	5.7 ± 1.8
Sex (M/F)	7/12	9/3	9/8	7/7
Onset (peracute/acute)	12/7	9/3	11/6	10/4
Onset duration^*^ (<12 h/12–24 h/>24 h) (*N* = 30)	14/4/0	6/5/1	11/5/0	9/4/1
Mean body weight (kg)	29.4 ± 18.4	22.1 ± 24.1	26.6 ± 15.5	28.7 ± 10.7
Median body condition score (1–9)	6 (4–8)	5 (4–9)	6 (4–9)	5 (4–7)
Neurolocalization (T3–L3/L4–S3)	13/6	12/0	13/4	12/2
Spinal shock^**^ (yes/no) (*N* = 23)	8/5	7/3	9/4	6/4
Schiff-Sherrington posture (yes/no)	8/11	6/6	8/9	6/8
Median PCSAL (%)	40 (26–88)	70 (39–80)	48 (26–88)	50 (26–79)
Median LL:VL ratio	3.2 (0.0–9.7)	1.4 (0.7–6.8)	1.4 (0.0–9.7)	3.4 (0.7–5.9)
Physiotherapy (yes/no)	3/16	6/6	4/13	5/9
Median duration of hospitalization (d)	6 (1–36)	5 (2–20)	6 (2–20)	5 (1–36)

Continuous variables presented as mean (± standard deviation) or median (range) as appropriate. ^*^Onset duration was unknown in 1 presumptive FCEM dog with pain perception. ^**^Spinal shock was assessed in the 23 dogs with T3–L3 only lesions on MRI. FCEM, fibrocartilaginous embolic myelopathy; ANNPE, acute non-compressive nucleus pulposus extrusion; PCSAL, percentage cross-sectional area of the lesion; LL:VL, lesion length to vertebral length.

25 dogs neurolocalized to T3–L3 spinal cord segments and 6 dogs localized to L4–S3 segments. Of the 17 dogs categorized as DPP, 11 dogs were DPP in both pelvic limbs and 6 dogs had pain perception in at least one pelvic limb toe or the tail base. Within those 17 DPP dogs, tail pain perception status was recorded in the medical record for 3 dogs. Two of these dogs had absent pain perception in their tail, while the remaining dog had positive tail pain perception with absent pain perception in both pelvic limbs. Of the 14 dogs classified as DPN, testing of tail base was recorded for 2 dogs and was negative. 9 of 31 dogs (29.0%) had pain on spinal palpation. Clinical signs compatible with spinal shock were detected in 15 of 23 dogs (65.2%) that had lesion on MRI affecting only the T3–L3 spinal cord segments. These clinical signs included decreased or absent withdrawal reflex of one or both of the pelvic limbs in 15 of 15 dogs (100.0%), decreased muscle tone of the pelvic limbs in 8 of 15 dogs (53.3%), decreased or absent patella reflex of one or both pelvic limbs in 6 of 15 dogs (40.0%), decreased or absent perineal reflex in 4 of 15 dogs (26.7%), decreased or absent anal tone in 3 of 15 dogs (20.0%), and decreased tail tone in 4 of 15 dogs (26.7%). Schiff–Sherrington posture was observed in 14 of 31 dogs (45.2%).

Based on MRI, the intramedullary lesion was affecting the T3–L3 spinal cord segments in 23 dogs, L4–S3 spinal cord segments in 1 dog, and both spinal cord segments in 7 dogs. 26 dogs had T1W post-contrast images for review; 1 of 26 dogs (3.8%) had a lesion that was minimally contrast enhancing. The median LL:VL ratio was 1.8 (0.0–9.7). The median PCSAL was 48.3% (26.0%–88.4%).

Median duration of hospitalization was 5 days (0–35 days). Twenty-eight dogs (90%) received medication during the hospitalization or at home after discharge. Medications included prazosin (*n* = 15), diazepam (*n* = 8), bethanechol (*n* = 2), tamusulosin (*n* = 1), gabapentin (*n* = 4), tramadol (*n* = 1), antibiotics (*n* = 11), prednisone (*n* = 1), and trazodone (*n* = 13). Outpatient physical rehabilitation was performed in 9 of 31 dogs (29.0%). The duration in 6 dogs ranged from 2 months to more than 2 years and was unknown in 3 dogs.

### Outcome and follow-up data

Follow-up information was available for 26 dogs including 2 dogs that were euthanized during hospitalization. Both dogs were DPN, and euthanasia was elected due to the severity of neurological status and owners’ concerns about prognosis. Five dogs (3 DPP, 2 DPN) were lost to follow up after discharge and had an unknown outcome. Excluding the 2 dogs euthanized during hospitalization, outcome could be assigned based on a recheck evaluation at the referral hospital in 9 dogs (7 successful, 2 unsuccessful) and was determined based on follow-up phone conversations with owners or referring veterinarians for 15 dogs (3 successful, 12 unsuccessful). Among the 26 dogs with information available, the median follow-up time from diagnosis to the time of last contact or death was 182 days (0–2,311 days). 10 of 26 dogs (38.5%) had a successful outcome. 1 of 10 dogs (10.0%) was DPN and the remaining 9 of 10 dogs (90.0%) were DPP on the initial neurological examination. 1 of the 10 dogs (10.0%) had persistent urinary incontinence described as frequent accidents inside home, and owners were manually expressing the bladder on a daily basis. 3 of the 10 dogs (30.0%), including the dog that also had urinary incontinence, had persistent, partial fecal incontinence, which was described as occasional accidents at home. 16 of 26 dogs (61.5%) remained non-ambulatory or were euthanized for reasons related to the presumptive diagnosis of FCEM or ANNPE and were classified as having an unsuccessful outcome. 11 of 16 dogs (68.8%) were DPN and 5 of 16 dogs (31.2%) were DPP on the initial neurological examination. Stratifying outcome by pain perception status, 1 of 12 DPN dogs (8.3%) had a successful outcome, while 9 of 14 DPP dogs (64.3%) had a successful outcome including 6 dogs with pain perception in both pelvic limbs and 3 dogs with pain perception in only one limb. The only DPN dog that had a successful outcome was a 6-year-old, male neutered Lagotto Romagnolo diagnosed with a presumptive ANNPE at the level of the 12th and 13th vertebrae. The dog regained pain perception in one limb 3 days after the onset, and subtle motor function was first noted on day 9. The dog participated in outpatient rehabilitation 4 times per week for 1 year. The dog eventually regained independent ambulation from 4 to 5 months after diagnosis and plateaued as an ambulatory paraparetic after approximately 1 year of the injury.

In addition to the 2 dogs that were euthanized around the time of diagnosis, 7 of 14 dogs (50.0%) with an unsuccessful outcome were eventually euthanized due to lack of improvement and concerns regarding quality of life. Median time to euthanasia in these 7 dogs was 27 days (7–180 days). 4 of 14 dogs (28.6%) were alive at the time of last contact (range 3–56 months) and 3 of 14 dogs (21.4%) were deceased between 2.7 and 4.7 years later due to reasons unrelated to the FCEM or ANNPE diagnosis.

### Logistic regression analyses

Univariable logistic regression analysis revealed that pain perception at presentation (*p* = 0.01), onset (*p* = 0.15), LL:VL ratio (*p* = 0.09), and spinal shock (*p* = 0.17) were potentially associated with outcome (*p* < 0.2; Table 2). These variables were incorporated into the multivariable logistic regression model (Table 3), which revealed that pain perception at presentation was the only factor associated with outcome. Dogs with absent pain perception were 47.40 times (95% CI: 2.09–1073.99%) more likely to have an unsuccessful outcome compared with dogs that had intact pain perception (*p* = 0.01). No other variables were identified as being associated with outcome (*p* > 0.05).

**Table 2:** Univariable logistic regression analysis of variables potentially associated with outcome in 26 dogs presumptively diagnosed with FCEM or ANNPE in which determination of outcome was possible.

Variables	Successful (*n* = 10)	Unsuccessful (*n* = 16)	OR (95% CI)	*p*-value
Mean age (y)	6.8 ± 3.1	6.0 ± 1.9	0.99 (0.70–1.20)	0.37
Sex (M/F)	6/4	7/9	0.52 (0.10–2.58)	0.42
Onset (peracute/acute)	9/1	10/6	5.40 (0.50–53.90)	0.15
Onset duration (<12 h/12–24 h/>24 h)	6/4/0	8/7/1	2.11 (0.45–3.61)	0.35
Diagnosis (FCEM/ANNPE)	4/6	11/5	2.20 (0.43–11.22)	0.34
Mean body weight (kg)	25.2 ± 15.4	29.5 ± 12.1	1.03 (0.96–1.09)	0.43
Median body condition score (1–9)	5 (4–8)	5 (4–9)	1.04 (0.54–2.01)	0.90
Pain perception (yes/no)	9/1	5/11	19.80 (1.94–201.62)	0.01
Neurolocalization (T3–L3/L4–S3)	9/1	13/3	2.08 (0.19–23.30)	0.55
Spinal shock (yes/no/NA)	8/1/1	6/4/6	0.19 (0.02–2.14)	0.18
Schiff-Sherrington (yes/no)	6/4	6/10	0.40 (0.08–2.01)	0.27
Median PCSAL (%)	48 (34–85)	48 (26–86)	0.17 (0.003–10.28)	0.40
Median LL:VL ratio	0.9 (0.0–3)	2.5 (0.0–9.7)	1.56 (0.93–2.67)	0.09
Physiotherapy (yes/no)	5/5	4/12	0.33 (0.62–1.79)	0.20
Median duration of hospitalization (d)	7 (4–20)	4 (0–36)	0.96 (0.87–1.07)	0.70

Continuous variables presented as mean (± standard deviation) or median (range) as appropriate. FCEM, fibrocartilaginous embolic myelopathy; ANNPE, acute non-compressive nucleus pulposus extrusion; PCSAL, percentage cross-sectional area of the lesion; LL:VL, lesion length to vertebral length; NA, not applicable.

**Table 3:** Multivariable logistic regression analysis of variables potentially associated with outcome in 26 dogs presumptively diagnosed with FCEM or ANNPE in which determination of outcome was possible.

Variables	Successful (*n* = 10)	Unsuccessful (*n* = 16)	OR (95% CI)	*P*-value
Onset (peracute/acute)	9/1	10/6	8.11 (0.32–204.91)	0.20
Pain perception (positive/negative)	9/1	5/11	47.40 (2.09–1073.99)	0.01
Median LL:VL ratio	0.9 (0.0–3.1)	2.5 (0.0–9.7)	1.22 (0.64–2.35)	0.55
Spinal shock (yes/no/NA)	8/1/1	6/4/6	0.18 (0.01–6.04)	0.34

Continuous variables presented as median (range). FCEM, fibrocartilaginous embolic myelopathy; ANNPE, acute non-compressive nucleus pulposus extrusion; LL:VL, lesion length to vertebral length; NA, not applicable.

## Discussion

In paraplegic DPN dogs presumptively diagnosed with FCEM or ANNPE, less than 10% of dogs recovered independent ambulation. This included 2 of 12 DPN dogs (17%) that were euthanized at diagnosis but were included in outcome analysis. In a previous report of dogs with FCEM, 3 of 7 (43%) DPN dogs that survived at least 1 week after diagnosis recovered ambulation. However, the recovery rate was 10% in that study if outcome included the 22 of 29 DPN dogs (76%) that were euthanized at or shortly after diagnosis or lacked follow-up information (12). Another study that investigated both FCEM and ANNPE included 10 DPN dogs, with 6 of 10 dogs (60%) euthanized at or shortly after the diagnosis. In the remaining dogs, 3 of 4 dogs (75%) regained ambulation (3). While our results appear lower for recovery in DPN dogs, direct comparison is challenging due to the high rate of euthanasia around the time of diagnosis in previous studies. Given the small case numbers, exclusion of these dogs in the evaluation of outcome might overestimate the recovery rate. Our results suggest that among dogs lacking pain perception secondary to presumptive FCEM or ANNPE, recovery of ambulation is rare.

In contrast to the dogs without pain perception, 64% of the paraplegic DPP dogs had a successful outcome. Most of these dogs had pain perception in both pelvic limbs at presentation, but several dogs also recovered where only one limb was DPP. In previous studies of FCEM and/or ANNPE, there is broad variation of reported success rates of regaining ambulation for paraplegic DPP dogs ranging from 45% to 96% (3, 7, 8). Among dogs from those studies and other previous studies that were less severely affected (i.e., non-ambulatory paraparesis), recovery rates were 87.5–100% (3, 7, 8, 11–13, 15). It is interesting to note that there is a similar success rate of approximately 60% for regaining ambulation with medical management in paraplegic DPP dogs secondary to TL-IVDE (14). While the prognosis appears to be fair to good for paraplegic DPP dogs with FCEM or ANNPE, our results indicate that a substantial proportion do not recover ambulation after their injury, potentially on par with medically managed TL-IVDE dogs. Due to small numbers, it was not possible to evaluate whether outcome differed between dogs with intact pain perception in both limbs and tail vs. pain perception only present in one of the limbs or tail.

Pain perception status at diagnosis was the only variable that was significantly associated with outcome. This finding supports that the absence of pain perception at presentation is a useful negative prognosic indicator in severely affected dogs with thoracolumbar presumptive FCEM or ANNPE. Similar trends have been reported in dogs with TL-IVDE. The success rate of DPP dogs has been reported to be 60% and 93% for medical and surgical treatment, respectively. Whereas for DPN dogs, it has been reported to be up to 21% and 61% for medical and surgical treatment, respectively (14). The absence of pain perception generally indicates a more severe spinal cord injury with a worse prognosis, and our data further support this in dogs with presumptive FCEM or ANNPE.

The extent of intramedullary hyperintensity on sagittal T2W images has been proposed as a prognostic indicator in various causes of spinal cord injury in dogs (7, 21–23). In our study, a significant relationship between LL:VL ratio and clinical outcome was not identified in multivariable logistic regression analysis. This is consistent with previous studies on ANNPE that did not find an association between LL:VL ratio and clinical outome (8, 24), in contrast to a study investigating clinical outcome in FCEM (7). The value of the LL:VL ratio in predicting clinical outcome in dogs with TL-IVDE is variable (21–23, 25, 26). Additionally, in our study, PCSAL was not associated with the clinical outcome of walking, whereas a previous study reported that PCSAL greater than 40% was associated with increased risk of urinary and fecal incontinence (13). These contradicting results likely reflect that while extensive T2W hyperintensity is indicative of more severe injury, the process of recovery from spinal cord injury is multifactorial, and it is difficult to predict reliably based on a single factor. Additionally, the present study only included severely affected dogs. If mild to moderately affected dogs were included that presumably would have had shorter LL:VL ratios, an association with outcome might have been found. Variation in magnetic field strength has also been suggested to impact the detection of intramedullary hyperintensity with higher field MRIs, especially at 3.0 Tesla, more frequently reporting no association between LL:VL ratio and clinical outcome (25, 26). The current study also used high field MRIs (1.5 or 3.0 T), which might have been another contributing factor to these results.

The longitudinal extent of T2W hyperintensity is also used as one of several MRI features in making a presumptive diagnosis of FCEM vs. ANNPE, with longer intramedullary hyperintensity supportive of FCEM (4, 15). Although the LL:VL ratio for the dogs with FCEM was nearly three times longer than that of the dogs with ANNPE, the difference was not statistically significant, and there was a large overlap between the two conditions. While a longer T2W intramedullary hyperintensity was utilized in this case series as one feature compatible with a diagnosis of FCEM, it is possible that the extent of the T2W hyperintensity may be less useful for differentiating presumptive diagnosis of FCEM and ANNPE in severely affected dogs.

In dogs with presumptive FCEM, 4 of 15 (27%) dogs had a successful outcome, whereas 6 of 11 (57%) dogs with presumptive ANNPE had a successful outcome. While the success rate was higher for ANNPE dogs, diagnosis did not significantly impact outcome. Previous reports comparing outcomes between these two conditions have higher but somewhat conflicting recovery rates ranging from 73% to 81.1% in presumptive ANNPE dogs compared with 67.4%–90% for presumptive FCEM dogs (3, 5). Direct comparison between studies and the two conditions is difficult for several reasons. Beyond the inherent challenge of ensuring the correct presumptive diagnosis, previous studies included dogs with variable neurological status and small numbers that were severely affected and had longer follow-up data available, whereas our data focused on only paraplegic dogs with or without pain perception. Additionally, the definition of a successful outcome was variable. In the current study, the focus was regaining ambulation, whereas other previous studies included recovery of urinary and/or fecal continence when considering outcome (3, 5). Our results indicate that outcomes are worse in severely affected dogs with either condition, but it remains unclear whether prognosis differs based on the specific diagnosis of FCEM or ANNPE.

The only DPN dog that had a successful outcome took months to recover walking and remained abnormal. This case highlights the importance of prolonged follow-up in dogs with severe spinal cord injury. A proportion of DPN dogs secondary to TL-IVDE managed surgically take more than 12 weeks to regain the ability to walk unassisted (27–29). Follow-up times in studies of dogs with FCEM and/or ANNPE suggest that there can be a similarly prolonged recovery of ambulation in some dogs (3, 5, 8, 12). 7 dogs with unsuccessful outcomes were euthanized due to their condition within 6 months of diagnosis, and 2 dogs (1 unknown and 1 unsuccessful outcome) had follow-up times of only 2 months and 3 months from the time of injury. It is possible that some of these dogs might have regained ambulation eventually if followed for a longer period of time.

The main limitations of the study are due to its retrospective nature and small sample size. Clinical outcomes were obtained via telephone follow-up with owners in the majority of the cases. This could have led to recall bias. However, the outcome measure was not complicated (ambulatory or not), which minimized the risk of this bias. Additionally, the medical record included pain perception status of the tail for only 5 dogs including 1 unsuccessful dog where the tail was DPP but the pelvic limbs were DPN. It is therefore possible that some DPN dogs where tail testing was not performed could have been misclassified (i.e., should have been in the DPP group), which could have impacted the proportions with a successful outcome in each group. None of the dogs had a definitive diagnosis of either FCEM or ANNPE by histopathological examination. Therefore, there is a possibility that the presumptive diagnosis of FCEM or ANNPE was misclassified as the other for some dogs. However, this represents a common clinical scenario, where a presumptive diagnosis of FCEM or ANNPE is based on compatible clinical findings and MRI characteristics. Due to the clinical similarity of the two conditions, we elected to consider them together, thus minimizing the impact of potential of misclassification of diagnosis on examination of outcome in dogs with either non-compressive myelopathy. Excluding the few cases that were euthanized or were lost to follow up shortly after diagnosis, no dogs demonstrated neurological deterioration, which supported the diagnosis of either FCEM or ANNPE and made alternative diagnoses unlikely. While this study does extend the information available for severely affected dogs, the small sample size underscores that recovery percentages should be interpreted with caution. Due to the small numbers, risk factors for persistent urinary or fecal incontinence also could not be assessed. Additionally, only one-third of the dogs participated in outpatient physical rehabilitation and the duration and extent of physiotherapy protocols were variable between individuals. Therefore, the effect of physiotherapy on the recovery from FCEM or ANNPE could not be evaluated. While pain perception status was the only variable associated with outcome, the substantial changes (>20%) in the odds ratios between univariable and multivariable analyses for both pain perception (positive or negative) and onset (peracute or acute) indicated that onset might be confounding the influence of pain perception on outcome. This was probably due to the small number of dogs with an acute onset and a successful outcome and suggested that the impact of onset could be worthy of further evaluation.

In summary, in paraplegic dogs diagnosed with presumptive thoracolumbar FCEM or ANNPE, the success rates for regaining ambulation for dogs with or without pain perception were 64% and 8%, respectively. Dogs lacking pain perception at diagnosis had a significantly higher risk of not regaining independent ambulation compared with the dogs with preserved pain perception. The absence of pain perception is a useful negative prognostic indicator in dogs with severe thoracolumbar FCEM or ANNPE, and such information could assist clinical decision-making.

## Data availability statement

The raw data supporting the conclusions of this article will be made available by the authors, without undue reservation.

## Ethics statement

Ethical approval was not required for the studies involving animals in accordance with the local legislation and institutional requirements because this was a retrospective study using medical record data only. Written informed consent was not obtained from the owners for the participation of their animals in this study because this was a retrospective study using medical record data only.

## Author contributions

GT: Writing – review & editing, Writing – original draft, Methodology, Formal analysis, Data curation, Conceptualization. ML: Supervision, Writing – review & editing, Writing – original draft, Methodology, Formal analysis, Data curation, Conceptualization. DD: Writing – review & editing, Writing – original draft, Methodology, Formal analysis, Data curation.
